# Hybrid Stent Implantation Into Right Ventricular Outflow Tract in a Newborn With Tetralogy of Fallot

**DOI:** 10.1016/j.jaccas.2023.102172

**Published:** 2023-12-21

**Authors:** Grzegorz Zalewski, Michał Buczyński, Michał Zawadzki, Paulina Kopacz, Jacek Kuźma

**Affiliations:** Cardiothoracic and Transplantology Department, Medical University of Warsaw, Warsaw, Poland

**Keywords:** anoxemic attacks, aortic coarctation, stents, tetralogy of Fallot

## Abstract

A case is presented of a newborn with tetralogy of Fallot and aortic coarctation. Progressive right ventricular outflow tract (RVOT) obstruction required urgent surgical therapy. Coronary artery crossing the outflow tract made ventriculotomy impossible. Hybrid RVOT stent implantation was performed, providing effective pulmonary flow and enabling postponing of corrective jumping graft implantation.

## Graphical abstract

**Figure:**
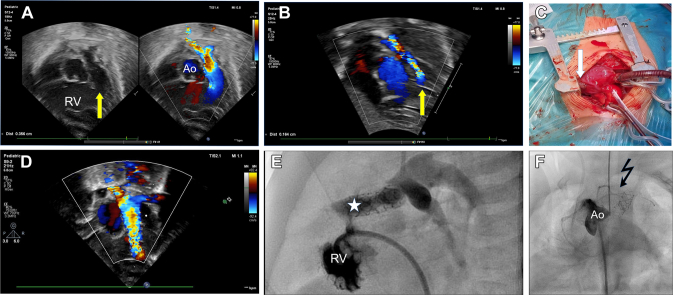

Tetralogy of Fallot (TOF) is a challenging cyanotic heart defect requiring early palliative cardiac surgery or invasive therapy (eg, right ventriculotomy with patch dilation, systemic-pulmonary anastomosis, or interventional RVOT stent implantation) in symptomatic neonates with other risk factors (eg, low body weight, hypoplastic pulmonary arteries, abnormal coronary arteries crossing the RVOT, coexisting genetic syndromes, and organ defects).^1^, ^2^, ^3^

A case is presented of a premature newborn of 35 weeks of gestation and birth weight of 1,850 g. Multiorgan congenital defects were diagnosed, including unilateral lip and palate cleft, additional 13th pair of ribs, butterfly vertebra, left preauricular processes, and a heart defect.

Transthoracic echocardiography (TTE) revealed complex TOF with aortic valve dextroposition of 50%, a large ventricular septal defect of anterior malalignment type, mild subpulmonary stenosis (Figure 1A), hypoplastic pulmonary valve annulus (5 mm, Z score −2.0), normal pulmonary and coronary arteries, and right aortic arch with isthmus stenosis, which extremely rarely coexists with TOF (Video 1, Video 2, Video 3, Video 4). Within 14 days, hypoxemic spells appeared, with oxygen saturation of 70% without improvement after propranolol therapy. Both TTE and CT showed severe progression of the subpulmonary stenosis (Figure 1B, Video 5), requiring urgent palliative surgery in cross-clamp circulation. Intraoperative evaluation of the coronary arteries revealed wide coronary arteries crossing the RVOT (Figure 1C), which made ventriculotomy impossible. Systemic-pulmonary anastomosis was an alternative procedure, but because of the infant’s low body weight of 2.3 kg, hybrid RVOT stent implantation was performed via a small ventriculotomy just below the additional left anterior descending artery. A Palmaz Genesis 6-mm × 18-mm stent was deployed into the RVOT under epicardial echocardiography guidance. TTE confirmed a stable stent position and effective pulmonary flow (Figure 1D, Video 6). The Postoperative course was uneventful, with a high SaO_2_ of 90%, and the child was discharged home. At the 2-month follow-up visit, the child’s condition was stable, with sufficient SaO_2_ (80%); however, owing to progressive stent stenosis (Video 7) and aortic coarctation as shown by TTE, with a pressure gradient of 50 mm Hg, heart catheterization was performed with effective balloon aortoplasty (Tyshak 8-mm balloon catheter), and the pressure gradient dropped to 5 mm Hg. RV angiography revealed severe stent obstruction by hypertrophied muscular bands (Figure 1E, Videos 8 and 9) leading to pulmonary flow impairment and progressive hypoxia. Aortography confirmed 2 wide coronary arteries crossing the RVOT (Figure 1F, Videos 10 and 11) and normal left coronary artery (Video 12). After dilation of the isthmus, the SaO_2_ dropped to 70%, and the child required a palliative systemic-pulmonary 3.5-mm anastomosis between the left subclavian artery and the pulmonary trunk (Video 13), which improved the SaO_2_ ≤85%. The patient is awaiting corrective therapy with jumping graft implantation and ventricular septal defect closure.

**Figure 1:**
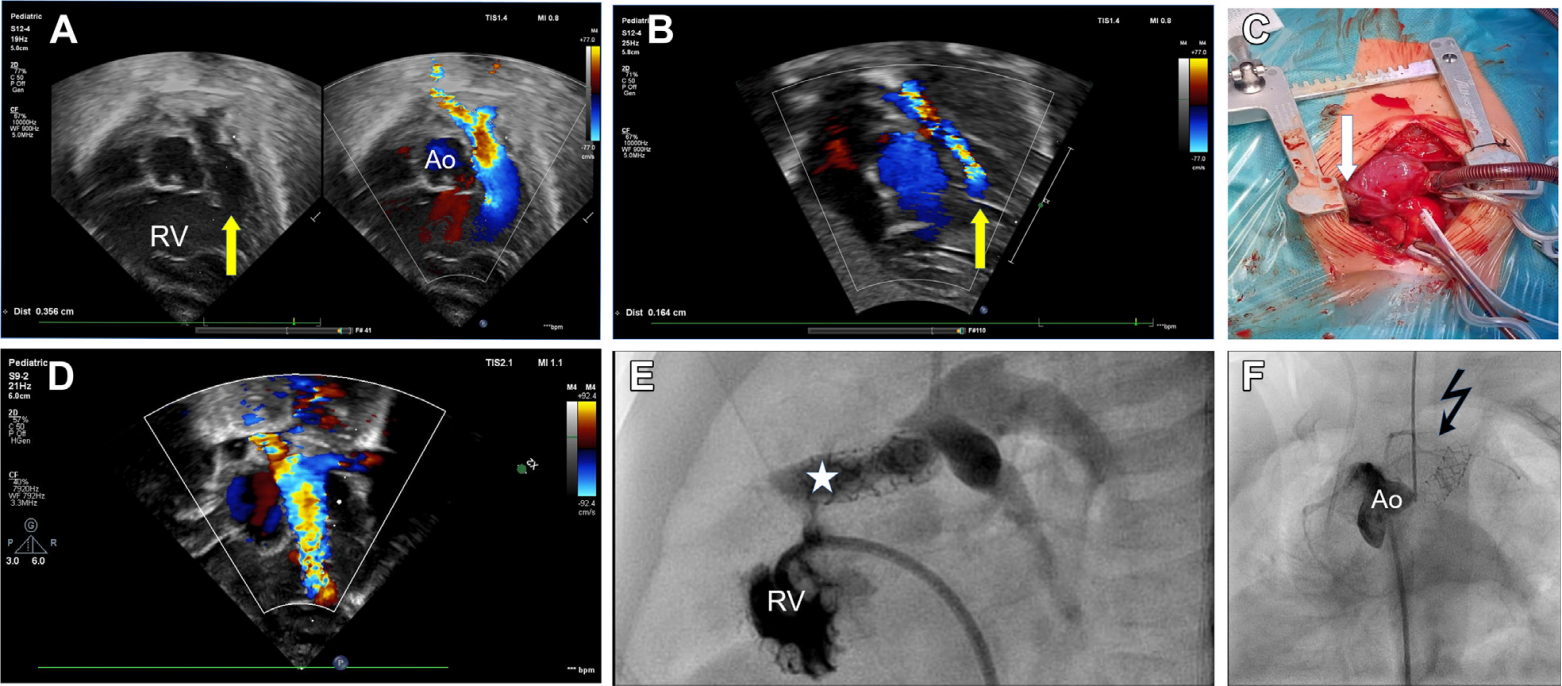
Progressive Right Outflow Tract Obstruction Despite Stent Implantation (A) Transthoracic echocardiogram (TTE). Subcostal view with color Doppler flow showing mildly stenotic right ventricular outflow tract (yellow arrow) on the first day of life with effective pulmonary flow. (B) TTE. Subcostal view with color Doppler flow showing severely stenotic right ventricular outflow tract (yellow arrow) impairing pulmonary flow. (C) Intraoperative view of coronary arteries with wide additional left anterior descending artery crossing outflow tract and a right ventriculotomy patch (white arrow) for hybrid procedure. (D) TTE. Subcostal view with color Doppler flow showing effective RV outflow tract flow through the implanted stent. (E) Right ventriculography in lateral view showing severe intraventricular stenosis by hypertrophied muscular bands obstructing the flow through the implanted stent (white star). (F) Aortography (CAU 40, CRA 30) showing right coronary artery, additional left anterior descending artery, and conal branch crossing outflow tract (dark jagged arrow). Ao = aorta; CAU = caudal; CRA = cranial; RV = right ventricle.

The authors conclude that careful follow-up observation of an infant with TOF is necessary because of the high risk of progressive RVOT obstruction with anoxemic spells. The staged palliative therapy is necessary in neonates with symptomatic TOF, low body weight, coexisting anomaly of the coronary arteries, and aortic coarctation. A hybrid approach with RVOT stent implantation is a safe and effective alternative solution in children with TOF anatomy unfavorable to other types of palliation (Supplemental Figure 1).

## Funding Support and Author Disclosures

The authors have reported that they have no relationships relevant to the contents of this paper to disclose.

## Appendix

Video 1 TTE. Parasternal longitudinal axis in 2D showing the aorta dextroposition and a large ventricular septal defect of malalignment type.

Video 2 TTE. Parasternal longitudinal axis with color Doppler flow showing large ventricular septal defect with left-to-right shunting.

Video 3 TTE. Subcostal view with color Doppler flow showing moderate right ventricular outflow tract obstruction on the 1^st^ day of life.

Video 4 TTE. High parasternal view with color Doppler flow showing right aortic arch and mild isthmus stenosis.

Video 5 TTE. Subcostal view with color Doppler flow showing severe subpulmonary obstruction in a 3^rd^ week of life.

Video 6 TTE. Parasternal view with color Doppler flow showing wide RVOT with Palmaz Genesis 6mm x 18mm stent deployed during the hybrid procedure.

Video 7 TTE. Parasternal view with color Doppler flow showing obstructed flow through the stent in RVOT at the age of 3 months.

Video 8 Right ventriculography in lateral view showing severe RVOT stenosis below and inside of the stent by muscular bands.

Video 9 Right ventriculography in antero-posterior view showing severe RVOT stenosis below and inside of the stent by muscular bands.

Video 10 Aortography in lateral view showing wide coronary arteries crossing RVOT, distended ascending aorta and isthmus stenosis.

Video 11 Aortography (CAU 40**°**, LAO 30**°**) showing RCA and coronary arteries crossing RVOT.

Video 12 Aortography (CAU 40**°**, LAO 30**°**) showing LCA with normal left anterior descending artery and circumflex artery.

Video 13 TTE. High parasternal view with color Doppler flow showing patent left sided Blalock Taussig shunt connecting left subclavian artery with pulmonary trunk.

Supplemental Figure 1 Hybrid Intraoperative Stent Implantation
