# Supplementary figures and images for: Hybrid Stent Implantation Into Right Ventricular Outflow Tract in a Newborn With Tetralogy of Fallot
Source: JACC Case Rep. 2023 Dec 21;29(3):102172. doi: 10.1016/j.jaccas.2023.102172 (PMC10865128; doi:10.1016/j.jaccas.2023.102172)

**Supplemental Figure 1**


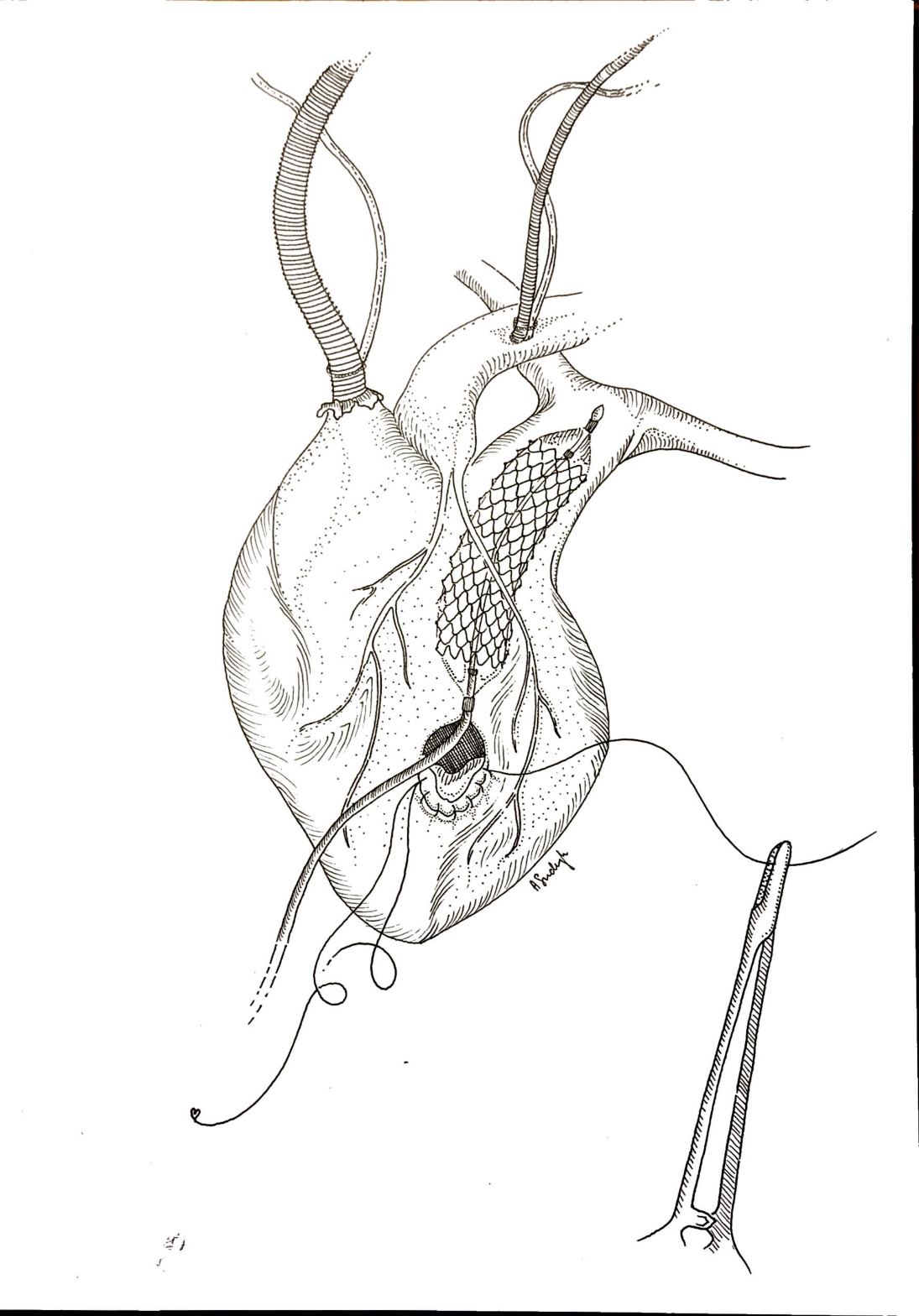

Supplement: Supplemental Figure 1 — Hybrid Intraoperative Stent Implantation [file mmc14.docx]
